# Influence of Mating Systems, Contemporary Niches, and Historical Demography on the Genetic Patterns of Flagship Plants From the Amazonian *Cangas*

**DOI:** 10.1111/eva.70325

**Published:** 2026-10-24

**Authors:** Bárbara Simões Santos Leal, Jacqueline Souza Lima, Lucas Erickson Nascimento da Costa, Carlos Eduardo Araújo Batista, Ana Carolina Galindo da Costa, Adriano Valentin‐Silva, Raquel Bezerra Chiavegatto, André Luiz de Rezende Cardoso, Lourival Tyski, Maurício Takashi Coutinho Watanabe, Valéria da Cunha Tavares, Carolina da Silva Carvalho

**Affiliations:** ^1^ Instituto Tecnológico Vale – ITV Belém Pará Brazil; ^2^ Programa de Pós‐graduação em Biodiversidade e Evolução Museu Paraense Emílio Goeldi Belém Pará Brazil; ^3^ Coordenação de Botânica Museu Paraense Emílio Goeldi Belém Pará Brazil; ^4^ Centro Nacional de Conservação da Flora ‐ CNCFlora Instituto de Pesquisas Jardim Botânico do Rio de Janeiro Rio de Janeiro Brazil; ^5^ Instituto de Estudos do Xingu, Universidade Federal do Sul e Sudeste do Pará São Félix do Xingu Pará Brazil; ^6^ Gerência de Recursos Naturais e Áreas Protegidas – Reserva Natural Vale Linhares Espirito Santo Brazil; ^7^ Bioparque Vale Amazônia Núcleo Urbano de Carajás Parauapebas Pará Brazil; ^8^ Mestrado Profissional em Uso Sustentável dos Recursos Naturais em Regiões Tropicais Instituto Tecnológico Vale – ITV Belém Pará Brazil; ^9^ Programa de Pós‐Graduação em Ciências Biológicas, Departamento de Sistemática e Ecologia Universidade Federal da Paraíba João Pessoa Paraíba Brazil

**Keywords:** comparative genetics, demographic responses, ironstone outcrops, mating system, population genomics

## Abstract

Genetic diversity is shaped by multiple factors, including life‐history traits, contemporary range distributions, niche constraints, and historical demography. The interplay of these drivers remains largely unexplored, particularly at scales proven to be relevant to conservation management of tropical biodiversity hotspots. Here, we investigate the relative contribution of each of these factors in shaping the current patterns of genetic diversity of 13 plant species occurring in Carajás, Southeastern Amazon, Brazil. We further test whether species associated with iron‐rich substrates in Carajás, known as *cangas,* share a common demographic history despite their differences in life‐history traits, distributions, and niches. We found that inbreeding levels are likely determined by mating systems, while both observed and expected heterozygosity were negatively associated with niche marginality. Surprisingly, distribution range and niche breadth had no effects on genetic parameters, likely due to the absence of correlations with ecological specialization. Demographic reconstructions revealed a lack of synchronous population histories across co‐distributed *cangas* plants, which likely reflects variation in the diversified life‐history traits affecting the timing of their responses to past climatic changes. Our results offer valuable insights into future demographic trends of *cangas*‐associated species, refining predictions on the fate of species in the face of anthropogenic pressures such as habitat loss and climate change. Our data further contribute to subsidizing analyses to determine priority areas and species for conservation.

## 1 Introduction

Understanding the determinants of genetic variation is crucial in evolutionary biology and conservation (Leffler et al. 2012; Ellegren and Galtier 2016), as genetic diversity underpins evolution and is essential for species adaptation and long‐term survival (Frankham 2005; DeWoody et al. 2021; Agashe et al. 2023). Species‐intrinsic characteristics, i.e., life‐history traits and the demographic history, shape genetic diversity through their effects on effective population size, mutation rates, and linked selection (Ellegren and Galtier 2016). In plants, reproductive traits such as mating systems, pollination modes, and seed dispersal have widely been recognized as major predictors of genetic diversity (Loveless and Hamrick 1984; Hamrick and Godt 1996; Duminil et al. 2007; Gamba and Muchhala 2020). Concurrently the demographic fluctuations driven by geological or climatic events play a key role in shaping genetic diversity in plants and animals (Hewitt 2004; Spielman et al. 2004; Theodoridis et al. 2020; Peart et al. 2020; Fonseca et al. 2023). Geographic range sizes have also been positively associated with genetic diversity (Cole 2003; Boyd et al. 2022), likely because widespread species occupy a broader environmental niche and experience greater environmental heterogeneity compared to narrow‐range specialists (Lira‐Noriega and Manthey 2014; Fine 2015; Szczecińska et al. 2016; De Kort et al. 2021). Indeed, a growing body of evidence highlights the influence of contemporary processes, life‐history traits, and historical demographic changes on genetic diversity patterns (Reusch et al. 2005; Pease et al. 2009; Pauls et al. 2013; Wernberg et al. 2018; De Kort et al. 2021). However studies integrating those multiple drivers of diversity are scarce (Romiguier et al. 2014; De Kort et al. 2021), particularly at spatial scales relevant to conservation management, such as at landscape and regional scales. Understanding the relative contribution of these drivers can guide conservation priorities and improve predictions for species with similar ecological and evolutionary profiles (Schoville et al. 2012), particularly when specific traits emerge as consistent predictors of genetic diversity.

To advance this understanding, comparative studies across co‐occurring taxa offer a promising avenue. There is growing recognition that taxonomically distinct species occupying the same region, and sharing habitat preferences and ecological requirements, are likely to have responded similarly to past environmental changes (Bermingham and Moritz 1998; Burney and Brumfield 2009; Papadopoulou and Knowles 2016). Nevertheless, demographic differences among such co‐occurring species are expected to be driven by variations in species‐specific traits, which influence how and when populations change in size and diverge (Zamudio et al. 2016). Thus, extending genetic analyses from single taxa to multiple co‐occurring taxa provides a valuable approach for estimating the impact and pervasiveness of past environmental changes (e.g., Buckley et al. 2022; Capurucho et al. 2023), thereby predicting the generality of species' responses to ongoing climate change. This approach is particularly informative when applied to habitat specialists, which tend to experience similar selection pressures due to their strong dependence on native habitats and restricted dispersal, often leading to concordant environmental responses (Kneitel 2018).

Terrestrial iron‐rich substrates offer a compelling context for exploring how co‐occurring species respond to shared ecological conditions. These substrates sustain ecologically unique ecosystems with a fragmented global distribution in regions such as northern and eastern Brazil, South Africa and western Australia (Gibson et al. 2010; Skirycz et al. 2014). Although some associated plants also occur in other environments, iron‐rich substrates typically harbor a high number of edaphic specialists (Corlett and Tomlinson 2020). Transitions between the iron‐rich substrate and surrounding matrix are often abrupt, resulting in island‐like habitats that isolate populations of habitat specialists. Indeed the comparatively harsher edaphic conditions found in iron‐rich substrates (i.e., nutrient‐poor, acidic soil with often poor water retention and mechanical support) limit plant communities, determining the high rates of plant endemism (Messias et al. 2013; Giulietti et al. 2019). The unique features of ironstone substrates may also influence the patterns of genetic diversity and differentiation within species. For instance, the patchy and disjoint distribution of iron‐rich substrates may constrain gene flow among populations (Ruas et al. 2020) and accelerate the effects of genetic drift within populations often resulting in low levels of genetic diversity and high inbreeding (Byrne et al. 2019; Leal et al. 2025). The relatively restricted area and high isolation among iron‐rich environments may also drive common demographic trends across species over time (Byrne et al. 2019). However, contrasting life history traits ultimately result in distinct responses to past and/or current environmental features, strongly influencing the patterns of genetic diversity (Romiguier et al. 2014; Papadopoulou and Knowles 2016; Brüniche‐Olsen et al. 2021). For instance, herbaceous plants, typically characterized by shorter life cycles, may exhibit greater resilience to past geoclimatic fluctuations, whereas shrubby taxa may retain stronger signatures of isolation or bottlenecks (Ossa et al. 2017).

The Carajás Mineral Province (hereafter Carajás), located in the southeastern Amazon, in Brazil, harbors some of the world's most prominent iron‐rich habitats, locally known as *cangas*. Unlike most iron‐rich ecosystems worldwide, the *cangas* plateaus in Carajás are surrounded by evergreen forests (Mota et al. 2018), creating a sharp ecological contrast and further reinforcing their insular character. Geologically, this region belongs to the Amazonian Craton, one of Earth's longest continuously exposed land surfaces (Shuster et al. 2012). Palynological records indicate that during warmer and wetter periods following Last Glacial Maximum (ca. 21. kya), evergreen forest expanded while *cangas* vegetation receded (Cordeiro et al. 2008; Guimarães et al. 2021; Reis et al. 2022). Such unique geological and climatic history of the Amazonian *cangas* underlies an astonishing plant diversity and endemism, which has been examined by comprehensive floristic studies (Mota et al. 2018; Giulietti et al. 2019; Andrino et al. 2020; Fonseca‐da‐Silva et al. 2020; Silva et al. 2024) as well as conservation‐applied population genetic studies (Lanes et al. 2018; Carvalho et al. 2019, 2021). Despite these efforts, there has been no attempt to integrate multispecies genetic data to evaluate, in a comparative framework, the major processes governing variation in species' genetic diversity in this Amazon ecosystem. Understanding whether life‐history traits and historical processes have differentially shaped genetic diversity across species can offer valuable insights into future demographic trends (Brüniche‐Olsen et al. 2021). Such insights are especially relevant given the intensifying anthropogenic threats facing the region, including mining expansion and climate change (see Schierenbeck 2017; Corlett and Tomlinson 2020), which is expected to reduce habitat suitability for most Carajás plants (Giannini et al. 2021).

Here, we investigate the relative influence of life‐history traits, contemporary distribution ranges and niches, and historical demographic changes in shaping current patterns of genetic diversity of 13 species from Carajás. Specifically, we address the following questions: (1) What is the relative importance of intrinsic species' features versus historical demographic processes in shaping current patterns of genetic diversity in plants from Carajás? (2) Do canga‐associated plants from Carajás share a common demographic history despite differences in their life‐history traits and ecological attributes? We hypothesize that contemporary ecological and life‐history characteristics exert a stronger influence on genetic diversity than past demographic changes (see Romiguier et al. 2014). Because these species occupy similar habitats within the same region, we predict that they may have experienced synchronous demographic responses to past environmental changes, including population size fluctuations and population splits. Beyond identifying the main drivers of genetic diversity in this iron‐rich ecosystem, our study contributes a comparative framework for prioritizing species for conservation and predicting vulnerability under ongoing anthropogenic pressures.

## 2 Material and Methods

### 2.1 Study Area

Carajás harbors a mosaic of forested and open vegetation, including dense evergreen forests and *cangas*. The area experiences high annual rainfall (~2000 mm) with pronounced seasonality—most precipitation occurs between January and March, while a dry season extends from June to August. Our study focused on four of the most prominent and well‐studied *canga* ridges in Carajás, ranging from 600 to 800 m in elevation: Serra Norte (SN), Serra Sul (SS), Serra do Tarzan (ST), and Serra da Bocaina (SB; Figure 1). SN and SS are located within the sustainable use reserve named Floresta Nacional (FLONA) de Carajás, which conciliates the conservation of pristine ecosystems and the exploitation of iron ore in both ridges. In contrast, ST and SB are part of the fully protected conservation unit named Parque Nacional (PARNA) dos Campos Ferruginosos. The combined area of the two conservation units is approximately 4910 km^2^—a land area comparable to that of some European countries. All four ridges are embedded within a matrix of evergreen forest typical of the Amazon biome (Figure 1).

**FIGURE 1:**
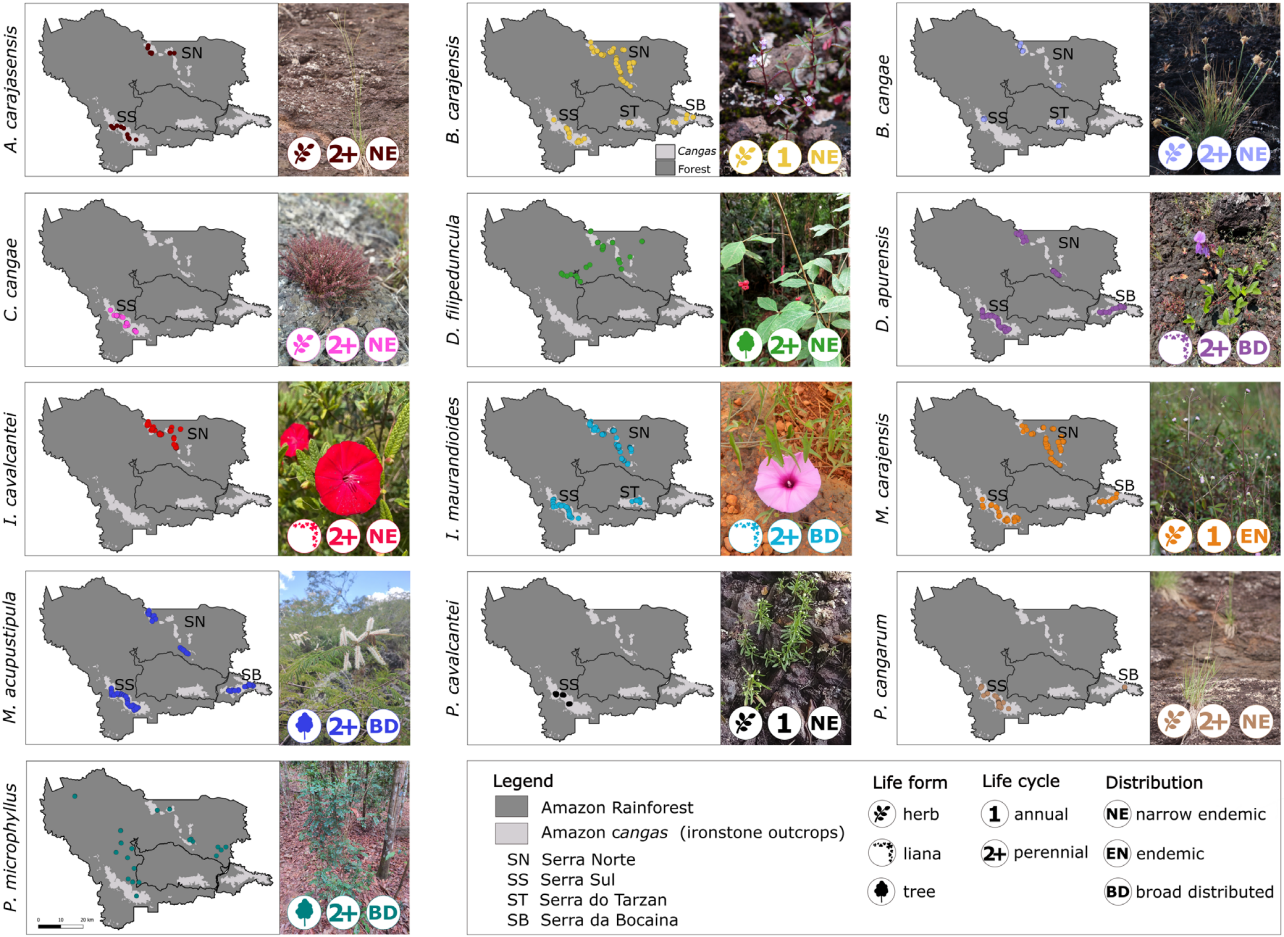
Life form, life cycle, distribution range, and location of sampled individuals of each studied species in the rainforest (dark gray) or in the *cangas* (light gray) of Serra Norte (SN), Serra Sul (SS), Serra do Tarzan (ST), and Serra da Bocaina (SB) within the FLONA de Carajás and PARNA dos Campos Ferruginosos. We considered species restricted to the limits of the study area (FLONA de Carajás and PARNA Campos Ferruginosos) as narrow endemics, those occurring within the study area and the surrounding region (Carajás) as endemics, and those distributed beyond Carajás as broadly distributed.

### 2.2 Studied Species

We evaluated genetic data from a total of 13 plant species occurring in Carajás. We re‐analyzed published SNP datasets obtained through reduced representation sequencing for nine out of 13 species: *Brasilianthus carajensis* Almeda & Michelangeli (Melastomataceae), *Carajasia cangae* R.M.Salas, E.L.Cabral & Dessein (Rubiaceae), *Dioclea apurensis* Kunth (Fabaceae), *Parapiqueria cavalcantei* R.M.King & H.Rob. (Asteraceae), *Ipomoea cavalcantei* D.F.Austin and *I. maurandioides* Meisn. (Convolvulaceae), *Monogereion carajensis* G.M.Barroso & R.M.King (Asteraceae), *Mimosa acutistipula* var. *ferrea* Barneby (Fabaceae), and *Pilocarpus microphyllus* Stapf ex Wardlew (Rutaceae) (Table 1, Figure 1). Additionally, we generated SNP data using the two enzyme genotyping by sequencing (teGBS; Poland et al. 2012) method for four other species occurring in this region: *Axonopus carajasensis* Bastos (Poaceae), *Bulbostylis cangae* C.S.Nunes & A.Gil (Cyperaceae), *Daphnopsis filipedunculata* Nevling & Barringer (Thymelaeaceae), and *Paspalum cangarum* C.O.Moura, P.L.Viana & R.C.Oliveira (Poaceae) (see Appendix S1 for a detailed description of the methods, Table 1, Figure 1).

**TABLE 1:** Characterization of the genomic data used in this study.

Species	Sampled sites	Number of individuals	Number of loci	Reduced representation method	Genetic data source
*Axonopus carajasensis*	*Cangas*: SN and SS	69	9496	teGBS	This study
*Brasilianthus carajensis*	*Cangas*: SN, SS, SB, and ST	131	500	nextRAD	Carvalho et al. (2019)
*Bulbostylis cangae*	*Cangas*: SN, SS, and ST	82	6049	teGBS	This study
*Carajasia cangae*	*Cangas*: SS	109	389^a^	GBS	Leal et al. (2025)
*Daphnopsis filipedunculata*	Forest	97	11,374	teGBS	This study
*Dioclea apurensis*	*Cangas*: SN, SS, and SB	163	2189	nextRAD	Carvalho et al. (2021)
*Ipomoea cavalcantei*	*Cangas*: SN	109	6944	nextRAD	Lanes et al. (2018)
*Ipomoea maurandioides*	*Cangas*: SN, SS, and ST	240	5252	nextRAD	Lanes et al. (2018)
*Mimosa acutistipula* var. *ferrea*	*Cangas*: SN, SS, and SB	172	1754	nextRAD	Carvalho et al. (2021)
*Monogereion carajensis*	*Cangas*: SN, SS, and SB	177	4032	nextRAD	Carvalho et al. (2019)
*Parapiqueria cavalcantei*	*Cangas*: SS	38	210^a^	GBS	Leal et al. (2025)
*Paspalum cangarum*	*Cangas*: SS and SB	69	8424	teGBS	This study
*Pilocarpus microphyllus*	Forest	289	8321	nextRAD	Monteiro et al. (2022)

Abbreviations: SB, Serra da Bocaina; SN, Serra Norte; SS, Serra Sul; ST, Serra do Tarzan.

^a^ For these species we filtered loci based on minimum allele count (mac = 2) instead of minimum allele frequency (maf = 2%).

Eleven out of the 13 studied species occur on the *cangas* within the study area, while two species are associated with forests surrounding the *cangas* (rainforest, Table 1, Figure 1). The species occurring on *cangas* were sampled in one to four distinct ridges (i.e., SN, SS, SB, and ST) according to the known distribution of each species. In contrast, *D. filipedunculata* and *P. microphyllus* were sampled throughout the forested matrix (Figure 1, Table 1). The studied species exhibit varying geographic ranges (broadly distributed to narrow endemic), growth forms (herbs, lianas, and trees), and life history strategies (annual to perennial; Table 1, Figure 1). All species are hermaphrodites, except for *D. filipedunculata*, which is cryptic dioecious (Watanabe et al. 2018; Carvalho et al. 2024). Details on the reproductive biology of each species are unknown, except for *I. cavalcantei*, which has been described as self‐incompatible and predominantly pollinated by hummingbirds (Babiychuk et al. 2019; Lanes et al. 2018; Table S1). Although we lack information on potential pollinators for the remaining species, previous studies classified them as wind‐ or insect‐pollinated based on their pollination syndromes (Pinto et al. 2020; see Table S1). Seed dispersal for most studied species is either abiotic (wind or water) or self‐driven (autochoric) as their seeds lack any structural adaptation to animal dispersal (see Table S1). Conversely, *D. filipedunculata* has fleshy fruits (berry‐like) and might be animal‐dispersed (personal observation, Table S1).

### 2.3 Datasets Filtering

To avoid among‐species bias in calculating the genetic parameters, we applied a standardized SNP filtering protocol to all datasets using vcftools (Danecek et al. 2011). From the initial variant datasets, we filtered one biallelic SNP per locus in Hardy–Weinberg equilibrium (*p* > 0.0001), with a minimum depth of 10 (‐‐minDP 10) and maximum depth (‐‐maxDP) corresponding to the mean + (5*SD). We dropped any variant with a minor allele frequency below two alleles (‐‐mac 2) for GBS datasets (see Table 1) or below the minimum frequency allele of 2% (‐‐maf) for teGBS, nextRAD, and genome skimming datasets. We also excluded SNPs with more than 30% missing data and individuals with more than 50% missing genotypes. Finally, we filtered out SNPs putatively under selection, as indicated by the outlier analysis using R package ‘pcadapt’ (Luu et al. 2017) under a *q*‐value threshold of 0.1. The number of retained SNPs per species is provided in Table 1. After all filtering procedures, the final datasets consisted of hundreds to thousands of high‐quality non‐linked SNPs (210 for *P. cavalcantei* to 11,374 SNPs for *D. filipedunculata*), with sample sizes varying from 38 to 240 individuals per species (in *P. cavalcantei* and *I. maurandioides*, respectively) (Table 1).

To ensure reliable inference of genetic parameters, we also assessed whether there was evidence of clonality and/or polyploidy in the studied species. For the species known (*D. filipedunculata*; Watanabe et al. 2018; Carvalho et al. 2024) or suspected to reproduce clonally via apomixis (i.e., *A. carajasensis* , *B. cangae*, and *P. cangarum*), we verified the presence of genetically identical individuals using the ‘mlg.filter’ function of R package ‘poppr’ (Kamvar et al. 2014, 2015). To do so, we set the Euclidean distance threshold specified by the ‘cutoff_predict’ function to differentiate clonal from non‐clonal samples. Clonality was confirmed only in *D. filipedunculata*, from which we removed 52 clonal samples, retaining 97 individuals for downstream analyses.

Given the frequent occurrence of polyploidy in Poaceae (Vaio et al. 2005; Bonasora et al. 2018; Goad et al. 2021), we also counted the chromosomes of *A. carajasensis* and *P. cangarum* using cytogenetic techniques. Both species exhibited chromosome numbers consistent with diploidy, i.e., twice the basic number (*x* = 10) as previously described for the clade within tribe Paniceae including both genera (*Axonopu*s and *Paspalum*, Giussani et al. 2001). For a detailed description of methods and results, see Appendix S2 and Figure S1.

### 2.4 Population Genetic Structure

To detect subpopulations within each studied species, we used a Bayesian approach implemented in the software Structure v2.3.4 (Pritchard et al. 2000). The analysis was run under the admixture model allowing for correlated allele frequencies, using a burn‐in of 20,000 followed by 100,000 Markov Chain Monte Carlo (MCMC) iterations. Ten replicate runs were performed per *K* with values of *K* ranging from 1 to 8. We inferred the best number of genetic clusters (*K*) for each studied species based on Evanno's method (Δ*K*) and the maximum likelihood (LnP(*K*)) using Structure Harvester v0.6.94 (Earl and VonHoldt 2012) and extracted individual admixture proportions for *K* = 2 to *K* = 4 averaged across runs using the main pipeline of the CLUMPAK server (Kopelman et al. 2015).

### 2.5 Genetic Response Variables

We calculated three response variables from the final datasets representing the genetic diversity within each species: observed heterozygosity (*H*_O_), expected heterozygosity (*H*_E_), and inbreeding coefficient (*F*_IS_), using the R packages ‘diveRsity’ (Keenan et al. 2013) and ‘hierfstat’ (Goudet 2005).

### 2.6 Ecological Predictors

We tested for the relative influence of life history, contemporary range distribution and niche, and historical demographic predictors on the interspecific variation of *H*_O_, *H*_E_, and *F*_IS_. As ecological predictors, we used (1) the pollen‐ovule ratio (PO ratio) – a proxy of the mating system in angiosperms (Cruden 1977), (2) the distribution range (DR) – as defined by the minimum convex hull polygon encompassing all known occurrences of each species, and (3) the niche marginality (NM) and breadth (NB) – measured using the outlying mean index (OMI) analysis, as described by Dolédec et al. (2000).

The PO ratio varies widely across angiosperms, reflecting the likelihood of pollen grains successfully reaching each stigma (Cruden 1977). Outcrossing species tend to produce more pollen grains per ovule to ensure successful fertilization, resulting in higher pollen‐ovule ratios than selfing species (e.g., Plitmann and Levin 1990; Alarcón et al. 2011). To estimate the PO ratios, we collected 10 pre‐anthesis flowers from different individuals collected within the study area and followed the method proposed by Cruden (1977). First, we dissected each flower to count the number of ovules under a stereomicroscope, and pollen was quantified from a single anther per flower. For this, the anther was macerated in distilled water on a slide under a stereomicroscope and covered with a gridded glass coverslip. Subsequently, we counted the pollen grains using an optical microscope. The total pollen production per flower was estimated by multiplying the anther count by the number of stamens. For *P. microphyllus* , due to the large amount of pollen, we counted grains from a single theca, doubled the count to represent the full anther, and then multiplied by the number of stamens. We obtained the PO ratio by dividing the total number of pollen grains by the total number of ovules in the same flower.

To calculate species' distribution ranges and niches, we first compiled occurrence records of each species from multiple sources, including the Global Biodiversity Information Facility (GBIF; http://gbif.org) and SpLink (http://specieslink.net) online databases, published literature and field surveys. After filtering the records for spatial accuracy and taxonomic consistency, we calculated the convex hull area using the ‘CHullAreaEarth’ function from the ‘GeoRange’ R package (Boyle 2017).

To estimate niche parameters, we used the OMI analysis, which combines principal component analysis with environmental conditions and species occurrence data. This method is widely adopted in ecological studies (e.g., Baastrup‐Spohr et al. 2015; Djordjević et al. 2016; Escoriza et al. 2021) due to its robustness to multicollinearity, independence from assumptions about species' response curves, and its capacity to effectively distinguish niche characteristics, even among closely related species (Thuiller et al. 2005; Djordjević et al. 2016). We first estimated the niche marginality (NM), which quantifies the distance between the average environmental conditions occupied by a species and the mean condition across the entire study area (Dolédec et al. 2000; Thuiller et al. 2005). Lower NM values indicate that a species occupies typical habitats within the environmental gradient, whereas higher values suggest that it occupies more distinct conditions of that environmental gradient (Dolédec et al. 2000; Farinas et al. 2015). Niche breadth (NB), in turn, reflects the range of environmental conditions a species is able to tolerate. High NB values indicate that the species can occur in a wide range of environmental conditions (i.e., generalists), whereas low NB values indicate that the species covers a narrower range of the environmental gradient (i.e., specialists, Thuiller et al. 2005; Farinas et al. 2015).

To retrieve environmental variables to the OMI analyses, we defined the study area within approximately a 100 km radius around the FLONA Carajás and PARNA Campos Ferruginosos. This radius was selected to encompass the core distribution of Amazonian *cangas*, including non‐sampled *cangas* sites in Carajas (i.e., Serra Leste, Serra Arqueada, and Serra de São Félix) and the Araguaia River basin, while maintaining a biologically relevant scale for regional environmental gradients. We initially selected 25 environmental predictor variables from multiple database: 19 bioclimatic variables with ~1 km resolution, from WorldClim 2 database (Fick and Hijmans 2017; https://www.worldclim.org/data/worldclim21.html), five topography variables with 30 m resolution from OpenTopography Facility (NASA Shuttle Radar Topography Mission (SRTM) 2013; https://doi.org/10.5069/G9445JDF) and the soil class at ~1 km resolution from AmbData (IBGE 2001; https://www.dpi.inpe.br/Ambdata/English/soil_map.php). To check for potential multicollinearity between the predictor variables, we calculated the variance inflation factor (VIF) using the ‘vif’ function from the ‘usdm’ R package (Naimi et al. 2014) and, excluded variables with a VIF greater than 5. We retained the following predictor variables: (1) mean diurnal temperature range (BIO02); (2) temperature seasonality (BIO04); (3) annual precipitation (BIO12); (4) precipitation of driest quarter (BIO17); (5) precipitation of coldest quarter (BIO19); (6) soil class; (7) aspect; (8) hillshade; (9) slope, and (10) digital elevation model (DEM) (Table S2).

### 2.7 Historical Predictor

As a historical predictor, we used the effective population size (*N*_E_) change, calculated as the difference between the estimated *N*_E_ during the LGM and that estimated for the Holocene period, providing a measure of demographic contraction and expansion over time. For this, we reconstructed changes in the *N*_E_ over time based on the site frequency spectrum (SFS) of each species using the Stairway Plot 2 program (Liu and Fu 2020). As recommended by the authors, we carried out the analysis using the two‐epoch model, defining 67% of variants for training. We used the easySFS script to convert SNP data into an unfolded SFS. Following its down‐projection approach, we evaluated different projection values using the ‐‐preview option and selected projections that maximized the number of segregating sites while retaining an adequate sample size for each population. We applied a rate of 10^−9^ base substitutions per site per generation (as described for *Arabidopsis thaliana* (L.) Heynh.; Ossowski et al. 2010) and adopted species‐specific generation time (i.e., age of first reproduction, as detailed in Table S1) based on expert knowledge and field observations.

### 2.8 Determinants of Genetic Diversity

Firstly, we examined the general covariation among genetic diversity predictors—i.e., PO ratio, distribution range, niche marginality and breadth, and *N*_E_ change—by estimating Spearman correlations with the ‘Hmisc’ and ‘corrplot’ packages. Significant correlations were found between niche breadth and distribution range, as well as between niche breadth and the PO ratio (Figure S2). To identify the main drivers of genetic diversity, we further tested the effect of each of these predictors on the genetic diversity parameters (*H*_O_, *H*_E_, and *F*_IS_). We employed single‐effect linear models (LMs) using the lme4 R package (Bates et al. 2015) to assess the influence of each predictor variable individually. Each model was compared to a reduced model without the predictor variable, using likelihood ratio test (LRT, *α* = 0.05), to test whether a model with a specific predictor differed significantly from a null model (i.e., intercept only). LRT were performed with the drop1 function in lme4 package. Given our limited sample size (13 species), we did not include more than one predictor per model and did not apply model selection procedures to avoid overfitting and inflated Type I errors.

### 2.9 Shared Demographic Patterns

We used GADMA (Noskova et al. 2020) to infer the demographic history of seven out of eleven *canga*‐associated species that occur on both Serra Norte and Serra Sul within the FLONA of Carajás: *A. carajasensis* , *B. carajensis, B. cangae, D. apurensis, I. maurandioides, M. acutistipula* var. *ferrea*, and *M. carajensis*. The remaining four species occurring on *cangas* were excluded from the analysis as they are restricted to either Serra Sul (*C. cangae*, *P. cavalcantei* and *P. cangarum*) or Serra Norte (*I. cavalcantei*). We did not include the remaining *cangas* sites (i.e., Serra do Tarzan and Serra da Bocaina) due to the limited number of species sampled in these areas. For each species, we constructed SFS using easySFS script, based on individuals sampled from Serra Norte and Serra Sul. GADMA was then used to automatically search for the best‐fit demographic model, assuming a two populations structure with one to two periods before and after the split. Consistent with the Stairway Plot 2 analyses, we applied a mutation rate of 10^−9^ substitutions per site per generation and adopted species‐specific generation times (Table S1). We additionally explored alternative demographic inferences using a mutation rate of 10^−8^ substitutions per site per generation to evaluate the sensitivity of model selection and parameter estimates to variation in mutation rate. For woody species (*D. apurensis*, *I. maurandioides*, and *M. acustitispula*), we also recalculated the timing of inferred demographic events assuming generation times twice as long as the values adopted in the main analyses to accommodate some uncertainty. For the remaining species, we assumed a generation time of 1 year, as they are either annuals (*M. carajensis* and *B. carajensis*) or fast‐growing graminoids ( *A. carajasensis* and *B. cangae*). We set moments (Jouganous et al. 2017) as the engine and used the L‐BFGS‐B method for local optimization. The best model for mutation rate in each species was selected using the Akaike Information Criterion (AIC).

## 3 Results

### 3.1 Population Genetic Structure

For most species, the *K* value identified by the Evanno method was consistent with the point at which LnP(*K*) reached a plateau (Figure S3). For some species, however, LnP(*K*) continued to increase toward higher *K* values that were not biologically plausible given the number of sampled localities. We therefore report the clustering resulting from *K* value identified by the Evanno method for each species and additionally show the inferred genetic structure for *K* = 2 to *K* = 4 (the maximum number of sampled localities), allowing a direct evaluation of the biologically relevant clustering patterns (Figure S4). The analysis revealed distinct subpopulations in Serra Sul (SS) and Serra Norte (SN) for all species but *B. carajensis* (Figure 2). For most species, individuals from each mountain ridge (SS, SN, ST, and SB) formed a single genetic cluster, despite some evidence of gene flow between ridges in *B. carajensis*, *B. cangae*, *D. apurensis*, *I. maurandioides*, *M. carajensis*, and *P. cangarum* (Figure 2, Figure S4). Except for *B. cangae*, individuals from the ST and SB mountain ridges either clustered with those from SN or formed distinct genetic clusters for the best *K* (Figure 2). These patterns of genetic differentiation among mountain ridges were observed either at the best K inferred by the Evanno method or at higher *K* values (see Figure S4). However, *B. carajensis*, *B. cangae*, and *I. cavalcantei* presented two distinct subpopulations in SN, whereas *P. cangarum* and *P. cavalcantei* showed two or three distinct subpopulations in SS (Figure 2). For *D. filipedunculata* and *P. microcephallus*, which occur throughout the forest matrix, the Evanno method inferred *K* = 3 and *K* = 2, respectively (Figure 2).

**FIGURE 2:**
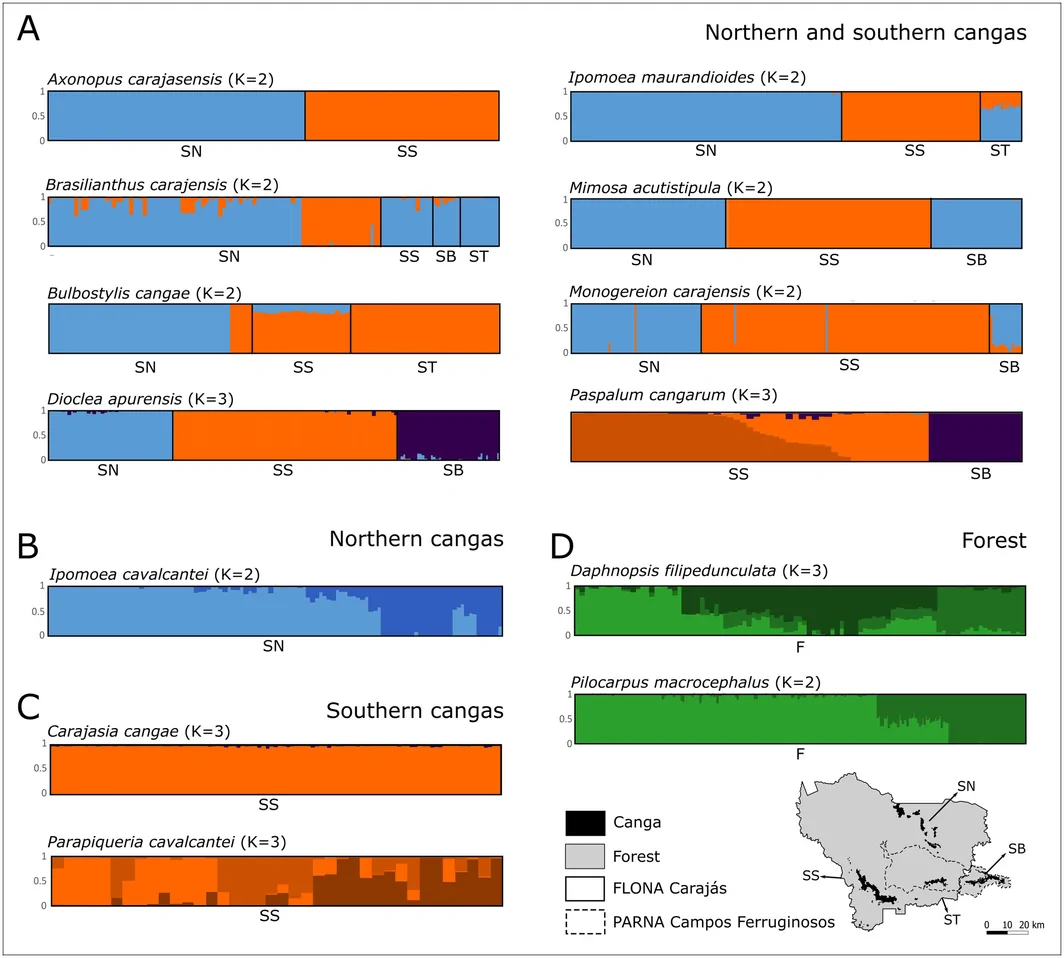
Patterns of genetic structure within each studied species as inferred by the Structure Bayesian analysis based on the final SNPs dataset. Species are organized by distribution as (A) species occurring in northern and southern *cangas*, (B) species restricted to the northern *cangas*, (C) species restricted to the southern *cangas*, and (D) species from forested areas.

### 3.2 The Role of Ecology and Demography in Shaping Genetic Diversity

Genetic parameters varied substantially among species, potentially reflecting differences in their ecological traits and demographic histories. Observed heterozygosity (*H*_O_) ranged from 0.004 in *C. cangae* to 0.193 in *B. carajensi*s, while expected heterozygosity (*H*_E_) showed variation from 0.016 in *C. cangae* to 0.193 in *M. acutistipula* var. *ferrea* (Table 2). *Carajasia cangae* exhibited an exceptionally high inbreeding coefficient (*F*_IS_ = 0.773) due to an excess of homozygotes, while *B. carajensis* had a negative *F*_IS_ (−0.039), suggesting an excess of heterozygotes (Table 2).

**TABLE 2:** Estimated genetic response variables and tested ecological and demographic history predictors.

Species	Genetic parameters			Ecological predictors				Historical predictor
	*H*_O_	*H*_E_	*F*_IS_	DR (km^2^)	NB	NM	PO ratio	*N*_E_ change
*Axonopus carajasensis*	0.100	0.117	0.147	530	0.572	3.217	3.724 ± 0.091	−78,939.6
*Brasilianthus carajensis*	0.193	0.186	−0.039	16,737	0.298	1.234	2.471 ± 0.049	−252,065.6
*Bulbostylis cangae*	0.110	0.127	0.132	5290	0.320	2.198	3.600 ± 0.140	−25,954.4
*Carajasia cangae*	0.004	0.016	0.773	108	0.046	6.022	2.161 ± 0.125	−3056.0
*Daphnopsis filipedunculata*	0.171	0.183	0.051	514	0.194	1.788	—	−340,389.2
*Dioclea apurensis*	0.147	0.164	0.106	2,200,842	1.046	2.258	3.162 ± 0.310	−400,445.3
*Ipomoea cavalcantei*	0.150	0.179	0.177	424	0.037	1.252	3.270 ± 0.108	−252,705.6
*Ipomoea maurandioides*	0.119	0.131	0.088	2,923,489	0.375	2.016	3.187 ± 0.055	−299,338.9
*Mimosa acutistipula* var. *ferrea*	0.173	0.193	0.103	130,773	0.979	2.067	3.507 ± 0.084	−103,666.4
*Monogereion carajensis*	0.126	0.127	0.007	8241	0.603	0.874	3.526 ± 0.041	−278,372.6
*Parapiqueria cavalcantei*	0.092	0.161	0.428	13	0.008	6.571	2.156 ± 0.186	−843,538.7
*Paspalum cangarum*	0.087	0.103	0.153	309	1.061	4.004	3.759 ± 0.052	−1,447,254.2
*Pilocarpus microphyllus*	0.157	0.180	0.137	289,911	1.189	2.134	3.619 ± 0.090	−117,639.7

Abbreviations: DR, distribution range; *F*_IS_, inbreeding coefficient; *H*_E_, expected heterozygosity; *H*_O_, observed heterozygosity; NB, niche breadth; *N*_E_ change, historical effective population size changes, calculated as the difference between the estimated *N*_E_ during the LGM and that estimated for the Holocene period; NM, niche marginality; PO ratio, pollen‐ovule ratio.

The results of the single‐effect models assessing the influence of DR, NM, NB, PO ratio, and *N*_E_ change on the genetic diversity parameters are summarized in Table 3. Niche marginality had a significant negative effect on both H_O_ and H_E_ (Table 3; Figure 3), indicating that species occupying more marginal environmental conditions tend to exhibit lower genetic diversity. For *F*_IS_, the PO ratio had a significant negative effect, while niche marginality had a significant positive effect (Table 3; Figure 3), suggesting that the mating system and ecological specialization play an important role in shaping inbreeding patterns. Interestingly, the LRT indicated that distribution range and *N*_E_ change also contribute meaningfully to the variation in *F*_IS_ across species (Table 3).

**TABLE 3:** Results of the models regarding the effects of distribution range (DR), niche marginality (NM), niche breadth (NB), pollen‐ovule ratio (PO ratio), and historical change in the effective population size changes (*N*_E_ change, calculated as the difference between the estimated N_E_ during the LGM and that estimated for the Holocene period) on genetic diversity parameters of 13 species from Carajás, Brazil.

Model predictor	Adj‐*R* ^2^	Estimate	SE	*p*
Observed heterozygosity				
DR	0.168	0.006	0.003	0.091
NM	0.570	−0.0215	0.005	0.002*
NB	−0.008	0.032	0.034	0.363
PO ratio	0.004	0.026	0.025	0.331
*N*_E_ change	−0.071	1.682e−08	3.716e−08	0.660
Expected heterozygosity				
DR	0.037	0.004	0.004	0.252
NM	0.256	−0.0154	0.007	0.044*
NB	−0.046	0.023	0.034	0.506
PO ratio	−0.053	0.017	0.026	0.518
*N*_E_ change	−0.090	2.564e−09	3.698e−08	0.946
Inbreeding coefficient				
DR	0.205	−0.028	0.014	0.068*
NM	0.699	0.101	0.018	0.0002*
NB	0.080	−0.196	0.137	0.180
PO ratio	0.312	−0.225	0.092	0.035*
*N*_E_ change	−0.090	1.262e−08	1.601e−07	0.939

*Note:* For each variable, the adjusted coefficient of determination (Adj‐*R* ^2^), the estimate (*ᵦ*), the standard error (SE), and the *p*‐value are shown. Significant *p*‐values were highlighted in bold. Likelihood Ratio Test (LRT) was performed to assess if each predictor variable significantly improved the model's log‐likelihood. Significance levels (*p* < 0.05) are highlighted with *.

**FIGURE 3:**
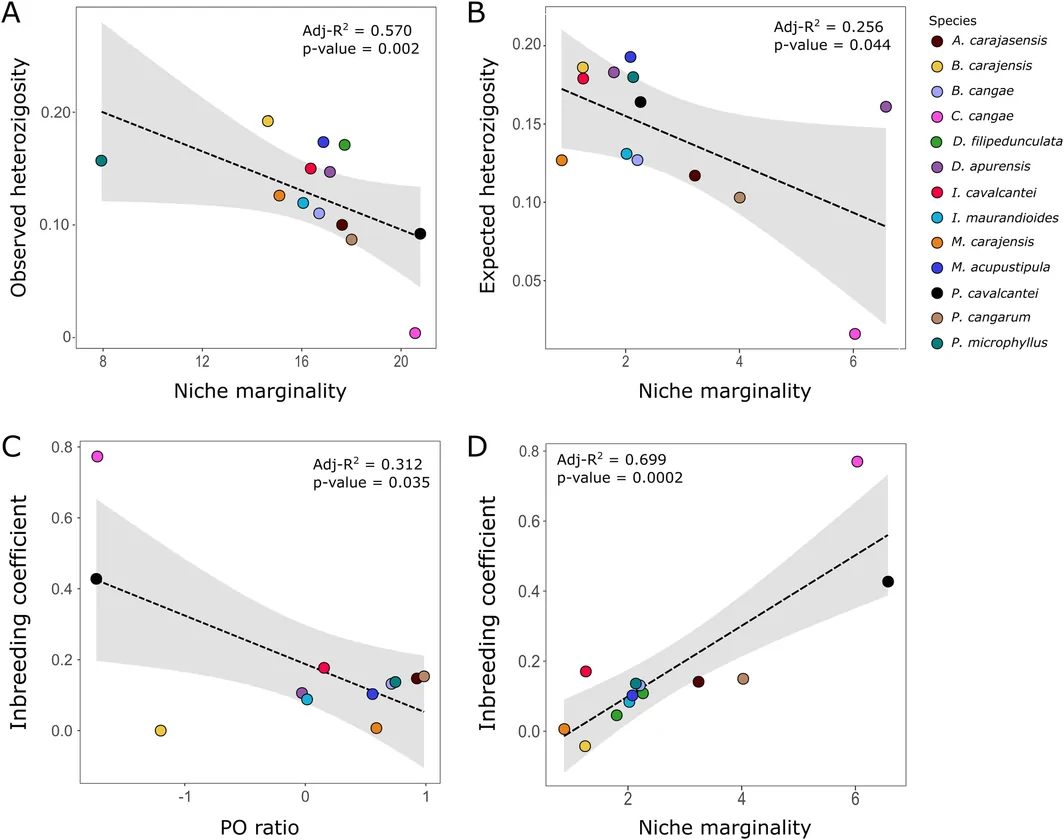
General effects of the niche marginality on the observed heterozygosity (*H*_O_; A), expected heterozygosity (*H*_E_; B), and inbreeding coefficient (*F*_IS_; C), as well as the effect of pollen‐ovule ratio on the *F*_IS_ (D) of 13 plant species occurring in Carajás, Brazil.

### 3.3 Historical Demography

The best‐fit demographic models inferred by GADMA with a mutation rate of 10^−9^ substitutions per site per generation suggest an asynchronous divergence during the Pleistocene (before the Last Glacial Maximum, except for *M. acutistipula* var. *ferrea*) between populations inhabiting Serra Norte (SN) and Serra Sul (SS) of each species (Figure 4). This pattern of asynchrony remained unchanged when generation times twice as long were assumed for the woody species (Figure 4H). Analyses using a mutation rate of 10^−8^ generally recovered similar demographic patterns (i.e., SS‐SN split, as well as contraction in both populations in most species, see Table S3) but placed the inferred events within the last few decades to a few thousand years ago for some species, an implausibly recent framework given the known geological history of the canga ecosystems (Shuster et al. 2012, see Table S3). We, therefore, present throughout the main text the estimates obtained under the 10^−9^ mutation rate. Most populations showed evidence of recent contraction, broadly consistent with the demographic patterns inferred by Stairway Plot 2. However, the population of *D. apurensis* from Serra Sul and both populations of *M. carajensis* exhibited signs of recent expansion or stability in population size throughout the Pleistocene (Figure 4). Bidirectional migration between SN and SS was observed for *A. carajensis*, *B. carajensis*, and *B. cangae*. For *M. acutistipula* var. *ferrea*, however, migration predominantly occurred from SN to SS, with migration from SS to SN restricted to the period immediately following the population split (Figure 4). Notably, the species for which no migration was inferred between SN and SS also showed no evidence of admixture in the genetic structure analyses. Conversely, the species with the most recent divergence between SN and SS exhibited detectable levels of admixture, consistent with their inferred history of gene flow (Table S3).

**FIGURE 4:**
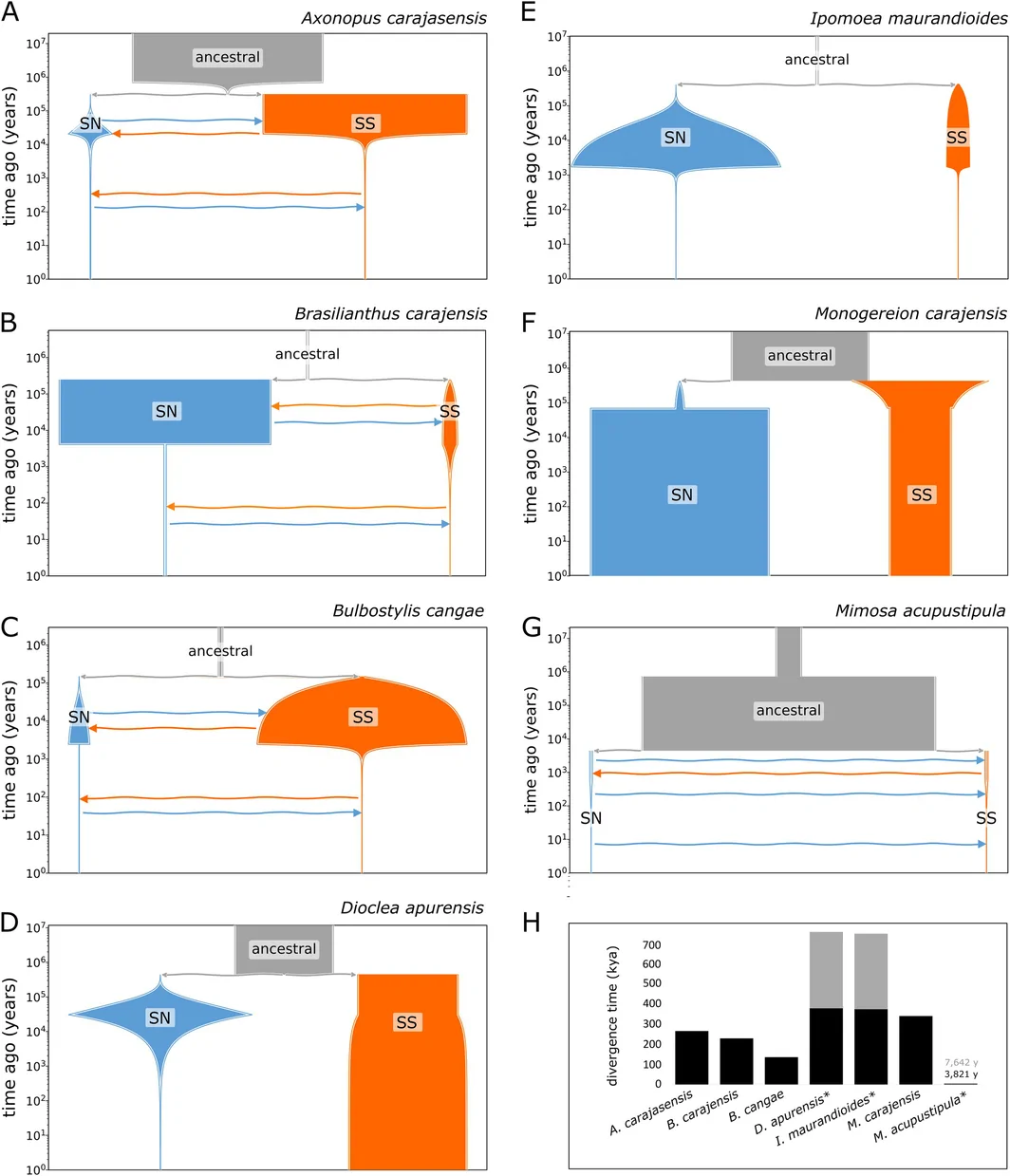
Best‐fit demographic models inferred using GADMA for the subset of seven species from the *cangas* of Carajás (A–G). Models were selected based on the Akaike Information Criterion (AIC) and reflect population dynamics over time (splits, bottlenecks, expansions, and migration) inferred from the site frequency spectrum data previously calculated for each species. Comparison among divergence times between Serra Norte (blue) and Serra Sul (orange) populations of each species (H). For woody species (marked with *), divergence times were estimated assuming two alternative generation times, and both estimates are displayed as black (shorter generation times) and gray (longer generation times) columns. SN, Serra Norte; SS, Serra Sul.

## 4 Discussion

Our study provides a comparative overview of genetic diversity and demographic history across 13 plant species in Carajás, southeastern Amazonia. Our results suggest that the inbreeding coefficient is shaped by species‐specific mating systems and niche marginality. The latter was also a strong predictor of patterns of observed and expected heterozygosity, highlighting an association between ecological specialization and genetic variation. These results indicate that contemporary ecological and life‐history characteristics explained more variation in genetic diversity than historical demographic processes. Moreover, we found no evidence for synchronous demographic events (e.g., population size fluctuations and splits) across the *cangas‐*associated flora. Together, these findings underscore the need to consider multiple ecological and historical factors when investigating the determinants of genetic diversity in co‐distributed plant species. Importantly, they reveal that different genetic parameters may respond to distinct drivers operating simultaneously (Ortego et al. 2012; Cheng et al. 2012; De Kort et al. 2021).

### 4.1 Interacting Factors Shape the Genetic Diversity in Carajás

We identified the pollen‐ovule ratio as one of the predictors of inbreeding rates among plant species inhabiting the *cangas*. High inbreeding rates can be both related to selfing reproduction and to the frequent occurrence of biparental mating among closely related individuals—a phenomenon commonly observed in small populations (Porcher and Lande 2016). Consistent with long‐established evidence linking mating systems to genetic patterns in plants (e.g., Loveless and Hamrick 1984; Hamrick and Godt 1996; Duminil et al. 2007; Gamba and Muchhala 2020), our findings highlight the overriding influence of life‐history traits on inbreeding patterns over historical demographic fluctuations (e.g., population size changes during the past 120 kya, corresponding to the Quaternary climatic oscillations). This underscores the need to conduct preliminary research on species biology to inform conservation efforts (Vähä et al. 2008). The lack of knowledge regarding key life‐history characteristics that influence genetic structure and diversity has posed significant challenges for effectively identifying and managing threatened species and populations (Dudash and Murren 2008), particularly in species‐rich tropical regions. Therefore, early research should prioritize collecting data on mating systems at the community level to identify potentially vulnerable species. Although high inbreeding rates are typically associated with increased inbreeding depression, the extent of this effect varies depending on the ecological context. For example, a meta‐analysis by Angeloni et al. (2011) showed populations that have remained small over long periods are less affected by inbreeding depression due to the purging of genetic load. Further research into the genetic basis of inbreeding depression and the efficacy of purging across different mating systems and life histories is, thus, essential to guide effective conservation strategies.

Our results also indicate that the inbreeding coefficient is driven by niche marginality, and secondarily predicted by distribution range, as models including this variable presented marginal significant *p*‐values and outperformed the null model. Interestingly, the association between niche marginality and inbreeding appears to be largely driven by the two predominantly selfing species, *C. cangae* (*F*_IS_ = 0.773) and *P. cavalcantei* (*F*_IS_ = 0.428). Both species are highly specialized and restricted to extreme microhabitats, such as exposed rock surfaces and shaded rock fissures, respectively (Leal et al. 2025). These species have likely maintained a consistently small and isolated population over time as they exhibited the lowest historical *N*_E_ changes among all studied species, and the current distribution is restricted to a single *canga* plateau, which may also contribute to their current genetic signature. These results support the distinct demographic and ecological trajectories of *C. cangae* and *P. cavalcantei*, highlighting the critical role of the mating system and ecological specialization in shaping the genetic patterns of plants.

Curiously, the expected interplay between mating system, niche specialization, and distribution range did not reflect on genetic patterns in our study. Specifically, we expected selfing species/populations to occupy marginal environments, display narrower niche breadth and geographic ranges, and exhibit reduced genetic diversity due to limited opportunities for outcrossing. In general, selfing species/populations occupy range margins or ecologically marginal sites where opportunities for outcrossing are limited (Barrett 2010), which likely reflects the greater colonization success of self‐compatible species (Baker 1955; Stebbins 1957; Pannell 2015). Although we found significant correlations between PO ratio, a proxy of the mating system, and niche breadth, and between the latter and distribution range, these relationships are likely more complex or species‐specific (see Park et al. 2018; Evans and Jacquemyn 2020).

Our study also revealed a negative relationship between niche marginality and genetic diversity, measured as observed (*H*_O_) and expected heterozigosities (*H*_E_). This suggests that species occupying specific habitat conditions—i.e., those deviating further from the average conditions of the study area—tend to harbor lower genetic diversity compared to species that are more centered within the environmental niche space (Lira‐Noriega and Manthey 2014; Gaytán‐Legaria et al. 2023). These results indicate that species restricted to marginal environments may be more prone to extinction under environmental changes, as lower genetic diversity can reflect reduced adaptive potential (Reed and Frankham 2003; Kawecki 2008; Capblancq et al. 2020). Conversely, species occupying more central portions of niche space are expected to cope with a broader range of environmental conditions, which could result in larger and more stable populations, sustaining higher effective population sizes (*N*_E_) and thus greater genetic diversity (Reed and Frankham 2003; Groot et al. 2011).

Interestingly, despite being positively associated with other predictors, niche breadth did not directly influence genetic parameters. Narrow ecological niches are usually associated with lower historical effective population sizes and more fragmented distributions than those observed in species with wider niche breadths, resulting in lower genetic diversity (Groot et al. 2011; Lira‐Noriega and Manthey 2014; Gaytán‐Legaria et al. 2023). Furthermore, niche breadth is often reported as an important predictor of species' range size (see Slatyer et al. 2013), as observed in our study. Our results underscore that niche breadth relationships might differ from the general expectations. Studies have demonstrated that general expectations regarding niche breadth responses may not be universal (Sexton et al. 2017). In the context of the Carajás region, we propose that the non‐significant responses of niche breadth could be linked to the environmental conditions of the *cangas*. Environmental extremes play a critical role in the evolution of niche breadth, and mechanisms driving niche breadth divergence in these habitats could involve selection for specialization under extreme conditions or niche shifts among species without concurrent changes in niche breadth (Hoffmann and Parsons 1993; Sexton et al. 2017). This framework might, for example, explain the lack of observed relationships between species niche marginality and breadth. While the mechanisms underlying niche‐genetic diversity relationships require further investigation, our results highlight the role of niche dynamics in shaping genetic diversity for species in Carajás. Specifically, they suggest that genetic diversity is likely influenced more by niche shifts than by niche expansion. From the conservation perspective, these patterns underscore the vulnerability of niche‐specialized species in Carajás, particularly in face of environmental changes driven by ongoing habitat loss from iron ore mining (Salles et al. 2019; Martins‐Oliveira et al. 2021) and the impacts of climate change (Giannini et al. 2021).

The distribution range, on the other hand, showed no significant effects on any genetic parameter. This result contrasts with some previous studies in which species' distribution range has been suggested as a potential predictor of genetic diversity (Gitzendanner and Soltis 2000; Szczecińska et al. 2016). Narrow distribution ranges are usually associated with lower genetic diversity (Fine 2015; Szczecińska et al. 2016), due to stronger effects of genetic drift and limited gene flow (Premoli et al. 2001; Nybom 2004). Meanwhile, widespread species can maintain high polymorphism and tend to be less impacted by genetic drift (Premoli et al. 2001; Nybom 2004; Szczecińska et al. 2016). However, in previous studies, the effect of distribution range is often associated with niche differences (Lira‐Noriega and Manthey 2014). In our study, no significant association was found between distribution range and niche marginality, which emerged as the main driver of genetic diversity. Such lack of correlation between geographic and niche parameters suggests that niche‐related processes—such as specialization and niche shifts—may play a more prominent role in shaping genetic diversity patterns (Lira‐Noriega and Manthey 2014; Alahuhta et al. 2017).

### 4.2 Non‐Concerted Demographic Responses of the Carajás Flora

The inferred demographic histories revealed differences in population dynamics between the Serra Norte and Serra Sul populations across species. This pattern remained robust when assuming a twofold longer generation time for woody perennial species or a faster mutation rate, resulting in similar or even greater asynchrony among demographic histories. Under mutation rates of 10^−9^ substitutions per site per generation, most species exhibited evidence of recent population contractions beginning approximately 1000 to 10,000 years ago. However, exceptions were observed in *D. apurensis* (Serra Sul subpopulation) and *M. carajensis* (both Serra Norte and Serra Sul subpopulations), which showed stable populations during the same time frame in which other species experienced demographic shifts (< 100,000 to < 10,000 years ago). These differences may reflect species‐specific ecological traits or adaptive capacities that enabled resilience or sustained growth despite regional climatic fluctuations. For example, *D. apurensis* and *M. carajensis* may exhibit traits or habitat preferences that mitigate environmental stressors, such as symbiotic nitrogen fixation capacity (Ramos et al. 2020). Furthermore, due to the superior stress resistance of symbiotic plants, many symbiotic nitrogen‐fixing plants serve as pioneers in colonizing and establishing vegetation in harsh environments given that they can grow on nitrogen‐poor soils (Ardley and Sprent 2021; Mathesius 2022). During periods of environmental change, such as climatic fluctuations or ecosystem disturbances, their competitive advantage may have allowed them to colonize and stabilize marginal habitats, promoting their establishment and range expansion into newly available or degraded ecological niches.

The broadly consistent demographic contraction patterns inferred by Stairway Plot 2 and GADMA for the other species may, in turn, reflect historical climatic fluctuations that drove shifts in habitat suitability and associated ecological pressures. During the Last Glacial Maximum (LGM), southeastern Amazonia experienced drier conditions compared to the Holocene, favoring the expansion of open vegetation over forest formations (Reis et al. 2017). However, post‐LGM climatic amelioration, particularly after the mid‐Holocene, led to substantial expansion of humid evergreen tropical rainforest, accompanied by a retraction of open vegetation (Reis et al. 2022). These transitions toward wetter environmental and climatic conditions may have contributed to the recent population declines detected for most species associated with the *cangas*. Looking forward, climatic projections for the Amazon suggest rising temperatures and decreasing precipitation (Nobre and Borma 2009; Gomes et al. 2019), conditions that may temporarily favor open‐vegetation‐adapted species. However, unlike past climatic shifts, future changes are expected to be more abrupt and extreme, likely surpassing the thermal tolerance limits of many native species (Sentinella et al. 2020). Coupled with ongoing deforestation, such rapid environmental shifts may overwhelm the adaptive or migration capacity of many taxa, leading to potential local extinctions.

## 5 Conclusions

Access to genomic data from multiple species has substantially advanced our understanding of the major processes shaping genetic diversity and enabled the identification of shared and divergent patterns among plant species from the Amazonian *cangas*. In this study, both the mating system and niche marginality emerged as key predictors of genetic diversity. While the inbreeding coefficient was primarily influenced by the mating system and niche marginality, heterozygosity levels were more closely linked to the latter. In contrast, historical demographic trajectories showed limited power to explain contemporary patterns of genetic diversity across species, suggesting that intrinsic biological traits and contemporary environmental conditions are stronger determinants of genetic variation in the canga flora. These findings reinforce the central role of genetic diversity as a predictor of fitness, adaptability, and, consequently, species resilience (Reed and Frankham 2003; Pease et al. 2009; Ortego et al. 2012). They also stress the need for an understanding of its drivers when developing effective management strategies and fostering the conservation of endangered species (Hedrick 2001; Sohn et al. 2013), including some of the species studied here. Moreover, our findings also revealed a lack of synchrony in demographic histories among co‐distributed plants inhabiting Serra Sul and Serra Norte, which likely reflects variations in multiple life‐history traits, such as generation time, reproductive strategies, and pollination and dispersal ability. These trait‐mediated responses likely modulate species' sensitivity to past and likely future environmental changes. Together, our results underscore the importance of integrating ecological and evolutionary perspectives to understand the mechanisms shaping genetic diversity and better assess species' vulnerability to both historical and projected climate dynamics. Such integration is key to guide conservation actions in biodiversity hotspots like *cangas*.

## Funding

This work was supported by Vale S.A., PEP R100603.PFC, PEP R100603.PLA.

## Conflicts of Interest

The authors declare the following financial interests/personal relationships, which may be considered as potential competing interests: all authors report that financial support was provided by Vale SA.

## Supporting information


**Appendix S1:** DNA extraction and library preparation.
**Appendix S2:** *Cytogenetics*.
**Figure S1:** Mitotic metaphases of (A) *Axonopus carajasensis* (2*n* = 2*x* = 20) and (B) *Paspalum cangarum* (2*n* = 2*x* = 20). Scale bar = 10 μm.
**Figure S2:** Correlation among tested predictors of the genetic diversity in 13 species of the Carajás Mineral Province. DR, distribution range; NB, niche breadth; NM, niche marginality; PO ratio, pollen‐ovule ratio; *N*_E_ change, difference between the estimated effective population size during the LGM and that estimated for the Holocene period.
**Figure S3:** Evaluation of the best number of genetic clusters (*K*) using LnP(*K*) and Delta *K*, as calculated with the Evanno method. Each panel (A–M) corresponds to one studied species and includes the variation in LnP(*K*) and Δ*K* across the tested *K* values (*K* = 1–8). The red vertical line indicates the *K* value selected according to the Evanno method.
**Figure S4:** Genetic structure within each studied species inferred by Bayesian analyses implemented in Structure, based on the final SNP datasets for *K* = 2 to *K* = 4. The studied species are distributed either in canga habitats or in forests within Carajás. F, Forest; SB, Serra da Bocaina; SN, Serra Norte; SS, Serra Sul; ST, Serra do Tarzan.
**Table S1:** Ecological characterization of each studied species in terms of life form, life cycle, sexual expression, dispersal syndrome, pollination syndrome, and habitat.
**Table S2:** Results of the Variance Inflation Factor (VIF < 5) of the ten selected predictor variables used in the outlying mean index (OMI) analysis. 15 variables from the 25 input variables have collinearity problems (VIF > 5): BIO01, BIO03, BIO05, BIO06, BIO07, BIO08, BIO09, BIO10, BIO11, BIO13, BIO14, BIO15, BIO16, BIO18, and roughness.
**Table S3:** Summary of the demographic history inferred for seven plant species from the Carajás cangas using GADMA under mutation rates of 10⁻⁹ and 10⁻⁸ substitutions per site per generation. The table summarizes the estimated divergence time between the Serra Norte (SN) and Serra Sul (SS) populations, inferred migration patterns, and inferred changes in effective population size.

## Data Availability

Raw sequencing data are deposited in the NCBI Sequence Read Archive (SRA, acession PRJNA1540789) and SNP datasets are available through the FigShare repository (https://doi.org/10.6084/m9.figshare.34717839).
