# Supplementary material for: Influence of Mating Systems, Contemporary Niches, and Historical Demography on the Genetic Patterns of Flagship Plants From the Amazonian Cangas
Source: Evol Appl. 2026 Oct 24;19(10):e70325. doi: 10.1111/eva.70325 (PMC13695023; doi:10.1111/eva.70325)
Supplement: Supplementary file 1 [file EVA-19-e70325-s001.docx]

**Supplementary Material**

**Supplementary appendix 1 -** *DNA extraction and library preparation*

We extracted genomic DNA from leaf samples of *Axonopus carajasensis* (N=69), *B. cangae* (N=82), *Daphnopsis filipedunculata* (N=163), and *Paspalum cangarum* (N=69) using the Plant DNeasy mini extraction kit (Qiagen). The quantification of DNA samples was performed using the dsDNA BR kit (Life Technologies), and two-enzyme GBS (teGBS) libraries were prepared following the protocol of Poland et al. (2012) with modifications. For each sample, we digested 7 µl of genomic DNA at 30 ng/µl-1 using the PstI and MseI restriction enzymes at 37°C for 12 hours. Then, we performed the ligation of Illumina adapters and barcodes at 22°C for 2 hours, followed by inactivation at 65°C for 20 min. The samples were pooled and purified using the QIAquick PCR Kit (Qiagen) and then enriched via PCR. We performed two additional purification steps using the QIAquick PCR purification kit (Qiagen) and AMPure XP beads (Beckman Coulter) to prevent the contamination of adapters and dimers. We checked the size of the DNA fragments using TapeStation (Agilent Inc.) and then carried out single-end sequencing (150 cycles) on four flow cells of the Illumina NextSeq 500 platform (Illumina Inc., San Diego, CA, USA).

We *de novo* assembled the raw data obtained through teGBS for each species separately using iPyrad (Eaton & Overcast 2020). For this, we set the parameters at default values, except clust_threshold (=0.95), filter_adapters (=2), and max_Indels_locus (=4).

**Supplementary appendix 2 -** *Cytogenetics*

We conducted the cytogenetic analyses using individuals of *A. carajasensis* and *P. cangarum* from Serra Sul (S11 plateau) under cultivation at the Instituto Tecnológico Vale, Belém, PA, Brazil. The root meristems underwent pre-treatment in 0.03% 8-hydroxyquinoline at 23°C for two hours, followed by storage at 6°C for 20 hours. After pre-treatment, we fixed the roots in Carnoy’s solution (3:1 absolute ethanol: glacial acetic acid) and stored them at -20°C until slide preparation. For enzymatic digestion, the root tips were treated in a solution containing 0.7% cellulase, 1% pectolyase, and 1% cytohelicase (Sigma-Aldrich) at 37°C for two hours. We prepared slides using the flame-drying technique described by Dong et al. (2000) and stained them with Fluoroshield containing DAPI. Finally, we captured photomicrographs of well-defined metaphase spreads using a Zeiss Axio Imager 2 epifluorescence microscope equipped with an Axioncam 506 color camera and ZEN Microscopy Software.

We identified 2n = 20 chromosomes in *A. carajasensis* and *P. cangarum* from mitotic metaphases of multiple accessions. These counts correspond to twice the basic chromosome number (x) reported for their respective genera (x = 10 for *Axonopus* and *Paspalum*), indicating that these species are diploid (Figure 1).

**Figure S1**. Mitotic metaphases of (A) *Axonopus carajasensis* (2n = 2x = 20) and (B) *Paspalum cangarum* (2n = 2x = 20). Scale bar = 10 µm.

**Figure S2.** Correlation among tested predictors of the genetic diversity in 13 species of the Carajás Mineral Province. DR = distribution range, NM = niche marginality, NB = niche breadth, PO ratio = pollen-ovule ratio, and N_E_ change = difference between the estimated effective population size during the LGM and that estimated for the Holocene period.

**Figure S3.** Evaluation of the best number of genetic clusters (K) using LnP(K) and Delta K, as calculated with the Evanno method. Each panel (A–M) corresponds to one studied species and includes the variation in LnP(K) and ΔK across the tested K values (K=1-8). The red vertical line indicates the K value selected according to the Evanno method**.**

**Figure S4.** Genetic structure within each studied species inferred by Bayesian analyses implemented in Structure, based on the final SNP datasets for K = 2 to K = 4. The studied species are distributed either in canga habitats or in forests within Carajás. Abbreviations: SN = Serra Norte, SS = Serra Sul, SB = Serra da Bocaina, ST = Serra do Tarzan, and F = Forest.

**Table S1.** Ecological characterization of each studied species in terms of life form, life cycle, sexual expression, dispersal syndrome, pollination syndrome, and habitat.

| **Species** | **Life form** | **Life cycle** | **Sexual expression** | **Generation time (years)** | **Dispersal syndrome** | **Pollination syndrome** | **Habitat: localities** |
| --- | --- | --- | --- | --- | --- | --- | --- |
| *Axonopus carajasensis* | herb | perennial | hermaphrodite | 1 | abiotic^A^ | anemophily^H^ | *canga*: SN and SS |
| *Brasilianthus carajensis* | herb | annual | hermaphrodite | 1 | abiotic^B^ | melitophily^G^ | *canga*: SN, SS, SB, and ST |
| *Bulbostylis cangae* | herb | perennial | hermaphrodite | 1 | anemochoric^C^ | anemophily^G^ | *canga*: SN, SS, and ST |
| *Carajasia cangae* | herb | bi-annual | hermaphrodite | 1 | abiotic/  autochoric^D^ | entomophily^H^ | *canga*: SS |
| *Daphnopsis filipedunculata* | shrubby tree | perennial | dioecious | 5 | zoochoric^A^ | melitophily^G^ | forest |
| *Dioclea apurensis* | liana | perennial | hermaphrodite | 3 | autochoric^E^ | melitophily^H^ | *canga*: SN, SS, and SB |
| *Ipomoea cavalcantei* | liana | perennial | hermaphrodite | 3 | anemochoric | ornitophily^H^ | canga: SN |
| *Ipomoea maurandioides* | liana | perennial | hermaphrodite | 3 | anemochoric | melitophily^H^ | *canga*: SN, SS, and ST |
| *Mimosa acutistipula* var. *ferrea* | shrubby tree | perennial | hermaphrodite | 5 | autochoric | melitophily^H^ | *canga*: SN, SS, and SB |
| *Monogereion carajensis* | herb | annual | hermaphrodite | 1 | abiotic^A^ | entomophily^H^ | *canga*: SN, SS, and SB |
| *Parapiqueria cavalcantei* | herb | annual | hermaphrodite | 1 | abiotic/  autochoric^F^ | entomophily^H^ | *canga*: SS |
| *Paspalum cangarum* | herb | perennial | hermaphrodite | 1 | anemochoric^C^ | anemophily^G^ | *canga*: SS and SB |
| *Pilocarpus microphyllus* | shrubby tree | perennial | hermaphrodite | 10 | autochoric^E,G^ | entomophily^G^ | forest |

**^A^**Giannini, T. C., Giulietti, A. M., Harley, R. M., Viana, P. L., Jaffe, R., Alves, R., ... & Siqueira, J. O. (2017). Selecting plant species for practical restoration of degraded lands using a multiple‐trait approach. Austral Ecology, 42(5), 510-521.

^B^Reginato, M., Vasconcelos, T. N., Kriebel, R., & Simoes, A. O. (2020). Is dispersal mode a driver of diversification and geographical distribution in the tropical plant family Melastomataceae? Molecular Phylogenetics and Evolution, 148, 106815.

^C^Munhoz, C. B. R., & Felfili, J. M. (2007). Reproductive phenology of an herbaceous-subshrub layer of a Savannah (Campo Sujo) in the Cerrado Biosphere Reserve I, Brazil. Brazilian Journal of Biology, 67, 299-307.

^D^Bremer, B., & Eriksson, O. (1992). Evolution of fruit characters and dispersal modes in the tropical family Rubiaceae. Biological Journal of the Linnean Society, 47(1), 79-95.

^E^Tavares, A. B., Bezerra, J. W. A., de Souza, F. G. L. S., da Silva, M. A. P., & Linhares, K. V. (2021). Síndromes de dispersão de espécies vegetais do cerrado sensu lato da Chapada do Araripe, Nordeste, Brasil. Pesquisas Botânica, 75, 155-195.

^F^personal observation

^G^Silva, C. E. P., Watanabe, M., Brito, R. M., Giannini, T. C. (2018) Sistemas de polinização nas cangas de Carajás. Produção Técnica ITV DS, Belém, Brazil.

**^H^**Pinto, C. E., Awade, M., Watanabe, M. T. C., Brito, R. M., Costa, W. F., Maia, U. M., ... & Giannini, T. C. (2020). Size and isolation of naturally isolated habitats do not affect plant-bee interactions: A case study of ferruginous outcrops within the eastern Amazon forest. PLoS One, 15(9), e0238685.

**Table S2.** Results of the Variance Inflation Factor (VIF < 5) of the ten selected predictor variables used in the outlying mean index (OMI) analysis. 15 variables from the 25 input variables have collinearity problems (VIF > 5): BIO01, BIO03, BIO05, BIO06, BIO07, BIO08, BIO09, BIO10, BIO11, BIO13, BIO14, BIO15, BIO16, BIO18, and roughness.

| **Variables** | **VIF** |
| --- | --- |
| Mean diurnal temperature range (BIO02) | 4.88 |
| Temperature seasonality (BIO04) | 2.45 |
| Annual precipitation (BIO12) | 4.77 |
| Precipitation of driest quarter (BIO17) | 1.89 |
| Precipitation of coldest quarter (BIO19) | 2.51 |
| Soil class | 1.56 |
| Aspect | 1.18 |
| Hillshade | 1.23 |
| Slope | 1.42 |
| Digital elevation model (DEM) | 2.03 |

**Table S3**. Summary of the demographic history inferred for seven plant species from the Carajás cangas using GADMA under mutation rates of 10⁻⁹ and 10⁻⁸ substitutions per site per generation. The table summarizes the estimated divergence time between the Serra Norte (SN) and Serra Sul (SS) populations, inferred migration patterns, and inferred changes in effective population size.

| **Species** | **Split SN-SS** | **Div time SN-SS** | **Migration SS > SN** | **Migration SN > SS** | **Pop change SS** | **Pop change SN** |
| --- | --- | --- | --- | --- | --- | --- |
| Demographic models with mutation rate of 10^-9^ | | | | | | |
| *Axonopus carajasensis* | Yes | 2.9 x 10^5^ | Yes | Yes | Retraction | Retraction |
| *Brasilianthus carajensis* | Yes | 2.5 x 10^5^ | Yes | Yes | Retraction | Retraction |
| *Bulbostylis cangae* | Yes | 1.5 x 10^5^ | Yes | Yes | Retraction | Retraction |
| *Dioclea apurensis* | Yes | 4 x 10^5^ to 8 x 10^5^ | No | No | Retraction | Stability |
| *Ipomoea marandioides* | Yes | 4 x 10^5^ to 8 x 10^5^ | No | No | Retraction | Retraction |
| *Monogeireon carajensis* | Yes | 3.7 x 10^5^ | No | No | Stability | Stability |
| *Mimosa acutistipula* var. *cangae* | Yes | 3.8 x 10^3^ to 7.6 x 10^3^ | Yes | Yes | Retraction | Retraction |
| Demographic models with mutation rate of 10^-8^ | | | | | | |
| *Axonopus carajasensis* | Yes | 1.6 x 10^4^ | Yes | Yes | Retraction | Retraction |
| *Brasilianthus carajensis* | Yes | 2.3 x 10^2^ | Yes | Yes | Stability | Stability |
| *Bulbostylis cangae* | Yes | 5.8 x 10^2^ | Yes | Yes | Discrete retraction | Retraction |
| *Dioclea apurensis* | Yes | 4.1 x 10^1^ to 8.2 x 10^1^ | Yes | Yes | Discrete expansion | Stability |
| *Ipomoea marandioides* | Yes | 3.7 x 10^4^ to 7.4 x 10^4^ | No | No | Discrete retraction | Retraction |
| *Monogeireon carajensis* | Yes | 4.3 x10^4^ | No | No | Retraction | Retraction |
| *Mimosa acutistipula* var. *cangae* | Yes | 4.7 x 10^3^ to 9.4 x 10^3^ | Yes | Yes | Retraction | Retraction |

**References**

Eaton, D. A., & Overcast, I. (2020). ipyrad: Interactive assembly and analysis of RADseq datasets. Bioinformatics, 36(8), 2592-2594.

Poland, J. A., Brown, P. J., Sorrells, M. E., & Jannink, J. L. (2012). Development of high-density genetic maps for barley and wheat using a novel two-enzyme genotyping-by-sequencing approach. PloS One, 7(2), e32253.

Dong, F., Song, J., Naess, S. K., Helgeson, J. P., Gebhardt, C., & Jiang, J. (2000). Development and applications of a set of chromosome-specific cytogenetic DNA markers in potato. Theoretical and Applied Genetics, 101, 1001-1007.
